# Altered Microstructure of Cerebral Gray Matter in Neuromyelitis Optica Spectrum Disorder-Optic Neuritis: A DKI Study

**DOI:** 10.3389/fnins.2021.738913

**Published:** 2021-12-20

**Authors:** Hanjuan Zhang, Qing Li, Lei Liu, Xiaoxia Qu, Qian Wang, Bingbing Yang, Junfang Xian

**Affiliations:** ^1^Department of Radiology, Beijing Tongren Hospital, Capital Medical University, Beijing, China; ^2^Department of Neurology, Beijing Tongren Hospital, Capital Medical University, Beijing, China

**Keywords:** neuromyelitis optica spectrum disorder, optic neuritis, DKI, microstructure, cerebral gray matter

## Abstract

The purpose of this study was to analyze microstructural alterations in cerebral gray matter using non-Gaussian diffusion kurtosis imaging (DKI) in neuromyelitis optica spectrum disorder (NMOSD) patients with optic neuritis (NMOSD-ON). DKI was performed in 14 NMOSD-ON patients and 22 normal controls (NCs). DKI-derived metrics, including mean kurtosis (MK), radial kurtosis (RK), axial kurtosis (AK), fractional anisotropy (FA), and mean diffusivity (MD), were voxel-wisely compared by two-sample *t-*tests with gaussian random field (GRF) correction between the two groups. The correlations between altered DKI metrics and clinical features were analyzed. Compared with NCs, NMOSD-ON patients showed significantly decreased MK and RK both in the left inferior temporal gyrus (ITG), and decreased AK in the bilateral calcarine (CAL). While increased MD in the left fusiform gyrus (FFG), right CAL, and right hippocampus (HIP)/parahippocampal gyrus (PHG) were found. Furthermore, correlation analysis showed that mean deviation was negatively correlated with AK values of bilateral CAL and positively correlated with MD values of right CAL (*q* < 0.05, false discovery rate (FDR) corrected). For NMOSD-ON patients, microstructural abnormalities in the occipital visual cortex are correlated with clinical disability. These findings may provide complementary information to understand the neuropathological mechanisms underlying the impairments of cerebral gray matter in NMOSD-ON.

## Introduction

Neuromyelitis optica spectrum disorder (NMOSD) is an inflammatory demyelinating disease mediated by inflammation against the aquaporin-4 (AQP4) water channel on astrocytes predominantly attacked optic nerves and spinal cord. For over half of NMOSD patients, optic neuritis (ON), characterized by decreased vision, pain during eye movement, and visual field defects (Vabanesi et al., 2019; Wu et al., 2019), is the first clinical feature (Oertel et al., 2018), which can trigger severe visual dysfunction accompanied by poor visual outcomes (Sotirchos et al., 2020). It has been reported that about three-fifths of NMOSD patients in the United States (Wingerchuk et al., 1999) were functionally blind in at least one eye, and nearly one-fifth of cases in the United Kingdom and Japan (Kitley et al., 2012) developed to permanent bilateral visual deficits.

Most neuroimaging studies of NMOSD have reported structural damage predominantly restricted to white matter, especially for optic radiation which is characterized by decreased FA and increased MD using diffusion tensor imaging (DTI; Yu et al., 2008; Jeantroux et al., 2012; Liu et al., 2012; Rueda et al., 2012; Zhao et al., 2012). The above findings are widely accepted as the mechanism of axonal degeneration secondary to ON. Subsequently, increasing studies have shown subtle morphological alteration in the visual cortex beyond the optic radiation, featured by reduced gray matter volume (Pichiecchio et al., 2012; Von Glehn et al., 2014; Liu et al., 2018) and cortical atrophy (Calabrese et al., 2012; Liu et al., 2014) in NMOSD patients. At present, anterograde *trans*-synaptic degeneration through the lateral geniculate nuclei has been considered as the leading mechanism to explain the reduced volume or thickness of the primary visual cortex in NMOSD patients with ON (Von Glehn et al., 2014; Manogaran et al., 2016; Tian et al., 2018), but the evidence for this mechanism remains inadequate.

Motivated by previous work, we performed diffusion kurtosis imaging (DKI) to explore the microstructural alterations of cerebral gray matter (cortex and/or subcortex) that likely precede detectable volumetric changes at the macroscopic level (Gong et al., 2017). On the one hand, DKI is a higher-order extension of DTI and provides a more accurate description of non-Gaussian diffusion of water molecules in heterogeneous and complex brain tissue than DTI that is a fundamental limitation given its assumption of Gaussian diffusion of biological water (Lu et al., 2006; Jensen and Helpern, 2010; Bester et al., 2015). On the other hand, DKI is sensitive to capturing the microstructural characteristics in isotropic tissues (gray matter) (Jensen et al., 2005), another limitation of DTI (Bester et al., 2015; McKenna et al., 2019). To date, DKI has been widely used in multiple diseases, such as schizophrenia (McKenna et al., 2019), Alzheimer’s disease (Xue et al., 2019), and normal tension glaucoma (Li et al., 2020). These findings imply a key role of DKI to investigate brain alteration.

Diffusion kurtosis imaging additionally provides three diffusion kurtosis tensor derived metrics, including MK, RK, and AK (Jensen and Helpern, 2010; Bester et al., 2015). MK serves as a measure of the overall kurtosis, suggesting the microstructural complexity and heterogeneity (Hui et al., 2008; Wu and Cheung, 2010). RK and AK are usually used to characterize the compactness of myelin and axon (Cheung et al., 2009; Kamagata et al., 2017). FA and MD, two commonly quoted metrics that also can be derived from DKI, are specific to assessing the integrity of myelin. Li et al. (2020) have reported that MK, AK, and FA were reduced in bilateral visual cortices in patients with normal tension glaucoma. Therefore, DKI may pave a new sight to deeper understand the neuropathology of NMOSD. In this study, we conducted a DKI study in NMOSD patients only with ON to characterize the altered microstructure in cerebral gray matter and its correlations with clinical features.

## Materials and Methods

### Subjects

A total of 21 NMOSD-ON patients and 22 NCs were enrolled in our study. The study was conducted following the Declaration of Helsinki, with ethical approval from the Beijing Tongren Hospital Committee. Written informed consent was obtained.

All patients enrolled in this study fulfilled the international consensus diagnostic criteria (Wingerchuk et al., 2015) for NMOSD with positive serum AQP4 immunoglobulin G antibodies (AQP4-IgG) and negative serum myelin oligodendrocyte glycoprotein immunoglobulin G antibodies (MOG-IgG). Only patients with ON, but without weakness of the limbs, were enrolled. The diagnosis of ON was made with the typical imaging findings of orbital magnetic resonance imaging (MRI) and typical clinical features. Within 1 week, Humphrey visual field exam was successfully performed to obtain mean deviation values. Age- and sex-matched NCs were recruited from the local community. All participants had to be right-handed. The exclusion criteria for both groups were as follows: (1) left-handed; (2) weakness of the limbs; (3) oculopathy and abnormalities detected in the optic pathway other than ON; (4) a history of other brain diseases, such as intracranial tumors, traumatic brain injury, and cranial surgery; (5) complications from other autoimmune disorders; (6) psychiatric disorders; (7) contraindications to MRI scanning; and (8) poor image quality. These exclusion criteria were mainly used to exclude the confounding results caused by other diseases or other factors.

In all, 14 NMOSD-ON patients and 22 NCs were finally included based on the inclusion/exclusion criteria. In the NMOSD-ON group, three patients were excluded due to the lack of Humphrey visual field test, and another three were excluded due to dyskinesia. One patient was excluded because of left-handedness.

### Magnetic Resonance Imaging Data Acquisition

Magnetic resonance imaging data sets were obtained with a 3.0-Tesla MR scanner (Discovery MR750; GE Healthcare, Milwaukee, WI, United States) with an 8-channel head coil. Spongy cushions were used to reduce head motion during the MRI examinations. DKI images were acquired using spin-echo single-shot diffusion kurtosis echo planar imaging. The parameters were as follows: TR = 6000 ms, TE = 70.8 ms, matrix = 128 × 128, field of view (FOV) = 256 mm × 256 mm; axial slices = 50; thickness = 3 mm; voxel size = 1 × 1 × 3 mm^3^; gap = 0 mm, flip angle = 90°, total acquisition time = 5 min and 36 s, *b* = 0/1000/2000 s/mm^2^. Five images were acquired at b = 0 s/mm^2^. Twenty-five direction images were acquired at b = 1000 and 2000 s/mm^2^ for each *b*-value.

### Data Preprocessing

The DKI data were preprocessed using Diffusion Imaging In Python (DIPY).^1^ The procedures were as follows: (1) The DICOM files of DKI were converted into NIFTI format; (2) The eddy current correction through the *eddy_correct*^2^ command in FSL was used to eliminate the distortion derived from head motion or eddy currents; (3) The b0 images were used to create a binary brain mask for brain extraction *via* the bet command in FSL^3^ (Smith, 2002); (4) The FA images of all subjects were aligned to a target FA images (FMRIB-58) in the Montreal Neurological Institute (MNI) space using a non-linear registration algorithm implemented in FNIRT (FMRIB3s Non-linear Registration Tool); (5) Other kurtosis and diffusivity (MK, RK, AK, and MD) images were registered onto the same space as the FA, while the computations were sped up and simplified through a cloud platform^4^ ; (6) Finally, all of the DKI-derived images were smoothed with a Gaussian kernel of 6 mm full-width at half-maximum (FWHM). The spatial resolution of the MNI space is 2 × 2 × 2 mm^3^.

### Statistical Analysis

#### Demographic and Clinical Data

Demographic and clinical data of all subjects were analyzed with the Statistical Package for the Social Sciences version 24.0 (SPSS, IBM Inc., United States). Shapiro-Wilk test was performed to analyze the normality for demographic and clinical data. The age and expanded disability status scale (EDSS) scores that followed non-normal distribution were shown as the median (range). The mean deviation that followed normal distribution was shown as the mean ± standard deviation. Group differences in age and sex data were analyzed using Mann-Whitney U test and Chi-square test, respectively. The significant threshold was set at *p* < 0.05.

#### Diffusion Kurtosis Imaging Data

Group comparisons of each smoothed DKI metric (MK/RK/AK/FA/MD) were carried out by DPABI software using two-sample *t*-tests withGRF correction (uncorrected voxel *p* < 0.001, corrected cluster *p* < 0.05). This correction was confined within gray matter mask. Age and sex were added as nuisance covariates. Furthermore, the linear correlation between mean values of altered DKI metrics in each cluster and mean deviation was assessed by Pearson correlation analyses. Multiple comparisons were corrected using aFDR method with a corrected threshold of *q* < 0.05.

## Results

### Demographics and Clinical Features

The clinical characteristics of the 14 NMOSD-ON patients and 22 NCs were listed in Table 1. There were no significant differences in age (*z* = −0.650, *p* = 0.532) and sex (χ^2^ = 0.156, *p* = 0.693) between the two groups.

**TABLE 1:** Demographic and clinical characteristics of NMOSD-ON and NCs groups.

	NMOSD-ON	NCs	*p*
Male/female	3/11	6/16	0.693^a^
Age, median (range) (y)	43.5 (22–55)	46.5 (23–60)	0.532^b^
Handedness (R/L)	14/0	22/0	NA
AQP4-IgG (±)	14/0	0	NA
Mean deviation, (mean ± SD) (dB)	−16.27 ± 9.27	0	NA
EDSS scores, median (range)	5 (2–6)	0	NA

*NMOSD-ON, neuromyelitis optica spectrum disorder-optic neuritis; NCs, normal controls; AQP4-IgG, serum aquaporin-4 immunoglobulin G antibodies; Mean deviation: an index of visual deficits derived from Humphrey visual field; EDSS, expanded disability status scale; NA: not applicable.*

*^a^The p-value was assessed by the Chi-squared test.*

*^b^The p-value was assessed by the Mann–Whitney U test.*

### Groups Differences in Diffusion Kurtosis Imaging Metrics

**FIGURE 1:**
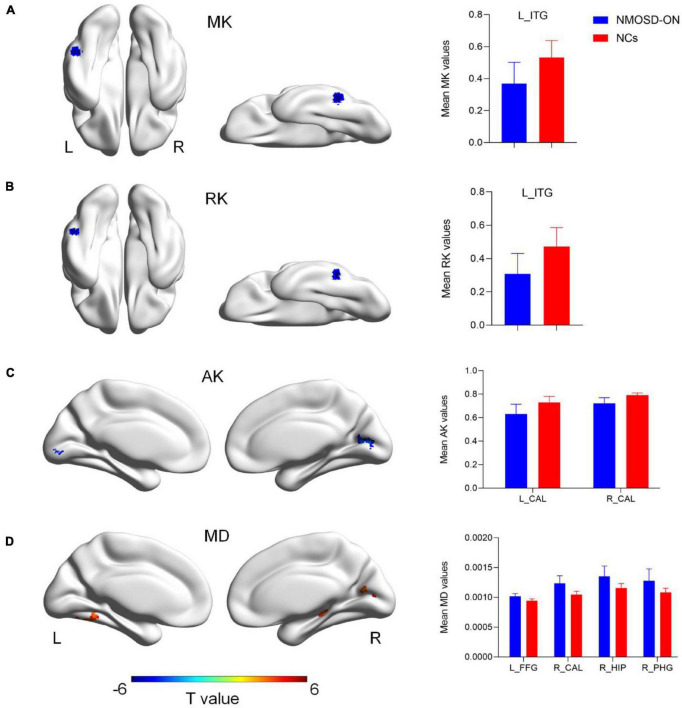
Diffusion kurtosis imaging metrics differences between the two groups (uncorrected voxel *p* < 0.001, corrected cluster *p* < 0.05, GRF corrected). **(A)** Compared with normal controls (NCs), NMOSD-ON patients showed decreased MK values in the left inferior temporal gyrus (ITG). **(B)** Decreased RK values were also observed in the left ITG. **(C)** Decreased AK values were observed in the bilateral calcarine (CAL). **(D)** Increased MD values were observed in the left fusiform gyrus (FFG), right CAL, right hippocampus (HIP)/parahippocampal gyrus (PHG). The details of the regions were shown in Table 2. NMOSD-ON, neuromyelitis optica spectrum disorder-optic neuritis; NCs, Normal controls; MK, mean kurtosis; RK, radial kurtosis; AK, axial kurtosis; MD, mean diffusivity; ITG, inferior temporal gyrus; CAL, calcarine; FFG, fusiform gyrus; HIP, hippocampus; PHG, parahippocampal gyrus; GRF, gaussian random field; L, left; R, right.

The results of the altered DKI metrics between the two groups were shown in Figure 1 (uncorrected voxel *p* < 0.001, corrected cluster *p* < 0.05, GRF corrected). Compared with NCs, NMOSD-ON group showed decreased MK and RK both in the leftITG (Figures 1A,B). AK was significantly decreased in the bilateral CAL (Figure 1C). MD was significantly increased in the left FFG, right CAL, and right HIP/PHG (Figure 1D). There was no difference in FA between the two groups. The details of the abnormal cerebral regions can be found in Table 2.

**TABLE 2:** Regions showing significant differences in DKI metrics between the NMOSD-ON and NCs groups.

Index	Brain regions	BA	Voxels	Peak MNI, mm			*t* score for peak Voxels
				*x*	*y*	*z*	
MK	Left ITG	20/37	130	−50	−50	−20	−4.4652
RK	Left ITG	20/37	160	−50	−50	−20	−4.8656
AK	Bilateral CAL	17/18	225	14	−90	6	−5.2414
MD	Left FFG	19/37	131	−32	−58	−18	4.5711
	Right CAL	17/18	151	10	−68	14	5.2600
	Right HIP/PHG	20/37	298	36	−30	−4	4.9035

*The statistical threshold was set at uncorrected voxel p < 0.001, corrected cluster p < 0.05, gaussian random field (GRF) corrected. The brain regions were vividly shown in Figure 1. MK, mean kurtosis; RK, radial kurtosis; AK, axial kurtosis; MD, mean diffusivity; ITG, inferior temporal gyrus; CAL, calcarine; FFG, fusiform gyrus; HIP, hippocampus; PHG, parahippocampal gyrus; BA, Brodmann’s area; MNI, Montreal Neurological Institute.*

### Significant Correlations Between Altered Diffusion Kurtosis Imaging Metrics and Clinical Features

**FIGURE 2:**
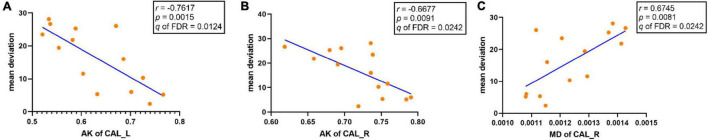
Significant correlations between altered DKI metrics and clinical features in the NMOSD-ON group (*q* < 0.05, FDR corrected). **(A)** AK of the left CAL was negatively correlated with mean deviation. **(B)** AK of the right CAL was negatively correlated with mean deviation. **(C)** MD of the right CAL was positively correlated with mean deviation. AK: axial kurtosis; MD, mean diffusivity; CAL, calcarine; FDR, false discovery rate; L, left; R, right.

Correlations between altered DKI metrics and the clinical features were shown in Figure 2. In the NMOSD-ON group, the mean AK values of bilateral CAL were negatively correlated with mean deviation (left CAL: *r* = −0.7617, *p* = 0.0015, *q* of FDR = 0.0124; right CAL: *r* = −0.6677, *p* = 0.0091, *q* of FDR = 0.0242). The mean MD values in the right CAL had a significantly positive correlation with mean deviation (*r* = 0.6745, *p* = 0.0081, *q* of FDR = 0.0242). There was no significant correlation between altered DKI metrics and EDSS scores in the NMOSD-ON group.

## Discussion

Compared with NCs, NMOSD-ON patients showed significantly decreased MK and RK both in the left ITG, and decreased AK in the bilateral CAL. While the abnormalities were found with the increased MD involving the left FFG, right CAL, and right HIP/PHG. There was no significant difference in FA. Moreover, correlation analysis showed that the mean deviation was negatively correlated with AK values of bilateral CAL and positively correlated with MD values of right CAL, showing the clinical implications of these findings in NMOSD-ON.

In the present study, significantly decreased kurtosis metrics (MK, RK, and AK) and increased MD were detected in the visual cortices of NMOSD-ON, including bilateral CAL, left ITG, and left FFG. Structurally, the visual pathway consists of the retina, optic nerve, chiasm, lateral geniculate nucleus, optic radiation, and visual cortex. The CAL of the occipital visual cortex is a part of Brodmann’s area 17 (BA17), BA18, and BA19. BA17, the primary visual cortex, and is densely connected with the optic nerve. BA18 and BA19 serve as the secondary visual cortex to integrate visual information received from BA17 and transmit it to the ventral and dorsal stream beyond the occipital lobe (Goodale and Milner, 1992). The ITG and FFG are the portions of the ventral stream where the decreased MK and RK and the increased MD suggested disability in visual stimuli processing, object recognition, and memory recall (Ishai et al., 1999; Haxby et al., 2000). In summary, the abnormal DKI metrics in these areas thus suggested that both primary and higher visual cortices are impaired. Several studies also have demonstrated the reduced volume or thickness in primary and higher visual cortices by voxel-based morphometry (VBM) and cortical analyses (Freesurfer) (Calabrese et al., 2012; Duan et al., 2012; Von Glehn et al., 2014; Wang et al., 2015; Tian et al., 2018). Beyond the abovementioned volumetric changes at the macroscopic level, we found cerebral cortical damage at the microscopic level. Decreased MK represents the reduced complexity of microstructure, resulting from neuronal edema, membrane structural damage, and myelin injury. Decreased RK and AK represent decreased compactness of myelination and fiber, while increased MD reflects the injury of myelin. As *trans*-synaptic degeneration is considered as the underlying mechanism of NMOSD (Von Glehn et al., 2014; Manogaran et al., 2016; Tian et al., 2018), we speculate that the microstructural damage in visual cortices could be attributed to it. Histologically, cortical lesions in NMOSD patients are characterized by the substantial loss of astrocytes, with secondary loss of either oligodendrocytes or neurons owing to astrocyte dysfunction and/or inflammatory damage (Kawachi and Lassmann, 2017; Jarius et al., 2020). Therefore, these DKI derived metrics characterized the microstructural damage likely attribute to secondary damage of the oligodendrocytes, and further support for the mechanism of *trans*-synaptic degeneration.

The HIP/PHG were found with the increased MD, consistent with the previous study (Liu et al., 2018). The HIP/PHG are subcortical structures that are parts of the limbic system and play a crucial role in cognitive function (Bush et al., 2000; Botvinick et al., 2004). Liu et al. (2015) have reported that the gray matter volume reductions in these regions may affect cognitive performance. As we didn’t perform neuropsychological assessment, there was no quantitative evidence to evaluate their cognitive problems. The microstructural alteration in these cognitive-related regions that are not directly connected with the visual cortex at least cannot be explained by *trans*-synaptic degeneration. We suspected that the bottom-up (from visual regions to cognitive-related regions) or top-down (from cognitive-related regions to visual regions) process could be the involved mechanism (McMains and Kastner, 2011). But the detail of the specific contribution needs to be clarified by future studies.

Mean deviation derived from Humphrey visual field is a commonly used index to evaluate visual field defects. We found that it was negatively correlated with AK values in the bilateral CAL and positively correlated with MD in the right CAL. The altered microstructure in these visual cortices in NMOSD-ON patients was mainly caused by anterograde *trans*-synaptic degeneration secondary to the damage of the optic nerve. These data thus suggest a potential role of microstructural damage in the visual cortices in predicting disability in NMOSD-ON patients.

## Limitations

Some limitations are present in this study. First, our findings are based on few patients. NMOSD is a rare disease, and it is difficult to perform large-scale studies, especially for patients with ON alone. Second, our findings are from a single imaging modality obtained in this sample. Recent studies have highlighted the importance of multimodal integration rather than the use of a single modality in revealing neural substrates underlying various brain disorders. In the future, we will conduct a multimodal study to further explore the alterations of cerebral gray matter among patients with ON, myelitis, or both ON and myelitis by cross-sectional designs. Finally, it is difficult to confirm whether these abnormalities are correlated with cognitive performance due to the lack of neuropsychological tests. We believe that this issue will be paid more attention.

## Conclusion

In summary, microstructural damage in the occipital visual cortex, correlating with visual deficits in NMOSD-ON patients, potentially acts as a substrate to explore the mechanisms underlying NMOSD-specific neurodegeneration. Moreover, the HIP/PHG beyond the visual cortex were involved, which may provide a novel insight into exploring its relationship with the loss of visual-related input.

## Data Availability Statement

The original contributions presented in the study are included in the article/supplementary material, further inquiries can be directed to the corresponding author.

## Ethics Statement

The studies involving human participants were reviewed and approved by Beijing Tongren hospital committee. The patients/participants provided their written informed consent to participate in this study.

## Author Contributions

HZ: conceptualization, methodology, formal analysis, investigation, writing original draft, writing review and editing, visualization, and funding acquisition. QL: data curation, conceptualization, methodology, formal analysis, investigation, visualization, and funding acquisition. LL: resources, data curation, conceptualization, and investigation. XQ: methodology, writing – review and editing, and visualization. QW: writing – review and editing and visualization. BY: visualization. JX: conceptualization, methodology, resources, supervision, project administration, and funding acquisition. All authors contributed to the article and approved the submitted version.

## Conflict of Interest

The authors declare that the research was conducted in the absence of any commercial or financial relationships that could be construed as a potential conflict of interest.

## Publisher’s Note

All claims expressed in this article are solely those of the authors and do not necessarily represent those of their affiliated organizations, or those of the publisher, the editors and the reviewers. Any product that may be evaluated in this article, or claim that may be made by its manufacturer, is not guaranteed or endorsed by the publisher.

## Footnotes

1
https://www.dipy.org/

2
http://fsl.fmrib.ox.ac.uk/fsl/fslwiki/EDDY

3
https://fsl.fmrib.ox.ac.uk/fsl/fslwiki/

4 www.humanbrain.cn, Beijing Intelligent Brain Cloud, Inc.
